# Evaluation of Polyphenol Anthocyanin-Enriched Extracts of Blackberry, Black Raspberry, Blueberry, Cranberry, Red Raspberry, and Strawberry for Free Radical Scavenging, Reactive Carbonyl Species Trapping, Anti-Glycation, Anti-β-Amyloid Aggregation, and Microglial Neuroprotective Effects

**DOI:** 10.3390/ijms19020461

**Published:** 2018-02-03

**Authors:** Hang Ma, Shelby L. Johnson, Weixi Liu, Nicholas A. DaSilva, Susan Meschwitz, Joel A. Dain, Navindra P. Seeram

**Affiliations:** 1 School of Chemical and Environment Engineering, Wuyi University; International Healthcare Innovation Institute (Jiangmen), Jiangmen 529020, Guangdong, China; hang_ma@uri.edu; 2 Bioactive Botanical Research Laboratory, Department of Biomedical and Pharmaceutical Sciences, College of Pharmacy, University of Rhode Island, Kingston, RI 02881, USA; shelby_johnson@uri.edu (S.L.J.); ndasilva@my.uri.edu (N.A.D.); 3 George and Anne Ryan Institute for Neuroscience, University of Rhode Island, Kingston, RI 02881, USA; 4 Department of Chemistry, University of Rhode Island, Kingston, RI 02881, USA; weixi_liu@my.uri.edu (W.L.); jdain@chm.uri.edu (J.A.D.); 5 Department of Chemistry, Salve Regina University, Newport, RI 02840, USA; Susan.Meschwitz@salve.edu

**Keywords:** berry anthocyanins, glycation, neuroprotection, beta amyloid, oxidative stress, Alzheimer’s disease

## Abstract

Glycation is associated with several neurodegenerative disorders, including Alzheimer’s disease (AD), where it potentiates the aggregation and toxicity of proteins such as β-amyloid (Aβ). Published studies support the anti-glycation and neuroprotective effects of several polyphenol-rich fruits, including berries, which are rich in anthocyanins. Herein, blackberry, black raspberry, blueberry, cranberry, red raspberry, and strawberry extracts were evaluated for: (1) total phenolic and anthocyanins contents, (2) free radical (DPPH) scavenging and reactive carbonyl species (methylglyoxal; MGO) trapping, (3) anti-glycation (using BSA-fructose and BSA-MGO models), (4) anti-Aβ aggregation (using thermal- and MGO-induced fibrillation models), and, (5) murine microglia (BV-2) neuroprotective properties. Berry crude extracts (CE) were fractionated to yield anthocyanins-free (ACF) and anthocyanins-enriched (ACE) extracts. The berry ACEs (at 100 μg/mL) showed superior free radical scavenging, reactive carbonyl species trapping, and anti-glycation effects compared to their respective ACFs. The berry ACEs (at 100 μg/mL) inhibited both thermal- and MGO-induced Aβ fibrillation. In addition, the berry ACEs (at 20 μg/mL) reduced H_2_O_2_-induced reactive oxygen species production, and lipopolysaccharide-induced nitric oxide species in BV-2 microglia as well as decreased H_2_O_2_-induced cytotoxicity and caspase-3/7 activity in BV-2 microglia. The free radical scavenging, reactive carbonyl trapping, anti-glycation, anti-Aβ fibrillation, and microglial neuroprotective effects of these berry extracts warrant further in vivo studies to evaluate their potential neuroprotective effects against AD.

## 1. Introduction

Glycation is a non-enzymatic process which starts with a reaction between sugar molecules and the free amino groups of proteins to produce unstable aldimine and ketoamine structures. Over time, these structures can then transform to a heterogenous group of compounds collectively known as advanced glycation endproducts (AGEs). Recently, it has been proposed that AGEs may contribute to the pathogenesis of several neurodegenerative diseases including Alzheimer’s disease (AD) [1]. In AD patients, significantly elevated levels of AGEs have been reported in neurons, astroglia, and cerebrospinal fluid [2]. Moreover, clinical studies have shown that subjects with higher circulatory levels of AGEs have a faster rate of cognitive decline [3]. 

Based on previous research findings, two major mechanisms have been proposed to link AGEs and AD, namely, (1) AGEs-induced oxidative stress, and, (2) reactive carbonyl species (RCS)-induced carbonyl stress [4]. Accumulated AGEs can magnify the production of reactive oxygen species (ROS) by binding to transmembrane receptors known as RAGE (receptor for AGEs), triggering intracellular signaling pathways, and stimulating the production of cytokines, which leads to neuronal oxidative stress, inflammation, and apoptosis. In addition, RCS, such as the active metabolic intermediate, methylglyoxal (MGO), can react rapidly with proteins to produce AGEs. These AGEs precursors exert direct cytotoxicity to neuronal cells by inducing the cross-linking of the beta amyloid (Aβ) protein and enhancing the formation of insoluble Aβ deposits, a pathological hallmark of AD. Moreover, ROS and RCS can also mediate cellular oxidative and inflammatory stresses, leading to the dysfunction of neuronal cells which greatly contributes towards the development of neurodegenerative diseases including AD [4,5]. 

Over the past years, our research group has been interested in the anti-AGEs effects of medicinal plants including plant foods and their derived extracts and pure compounds [6,7,8,9,10,11]. As part of this research program, we proposed an algorithm to predict the potential neuroprotective effects of traditional Ayurvedic medicinal plant extracts against AD using anti-glycation, anti-Aβ-fibrillation, and anti-neuroinflammatory bioassays [12]. In that project, we reported that the medicinal plant extracts which contained a high phenolic content, and showed free radical scavenging, RCS trapping, anti-glycation, anti-Aβ-fibrillation, and anti-neuroinflammatory properties were promising candidates for AD based research [12]. Given these observations [12], herein, we sought to similarly evaluate six common edible berries, which are particularly rich in polyphenols, including anthocyanins, in this panel of bioassays. Anthocyanins are the water-soluble pigments responsible for the colors of berries and have been extensively studied and implicated in the numerous biological effects and potential health benefits of these fruits [13]. 

Berry fruits have been reported to show anti-glycation [14] and potential neuroprotective properties [15,16]. Several wild berries, collected from northern Quebec in Canada, were recently reported to show anti-glycation activities which correlated with their antioxidant activities and total phenolic contents [17]. However, to date, there are limited studies linking the anti-glycation effects to the potential neuroprotective properties of common edible berries, namely, blackberry (*Rubus* sp.), black raspberry (*Rubus occidentalis*), blueberry (*Vaccinium angustifolium*), cranberry (*Vaccinium macrocarpon*), red raspberry (*Rubus idaeus*), and strawberry (*Fragaria ananassa*). The phenolic composition, including the identification of the individual anthocyanins in these berries have been extensively investigated [18,19,20,21,22,23]. Moreover, studies have shown that the synergistic, additive, and/or complementary effects of multiple constituents within whole plant foods and their extracts show biological effects greater than any single constituent alone [24]. Therefore, taking this “whole food/extract” approach, herein, we aimed to evaluate the aforementioned berry extracts (crude extracts, CEs; anthocyanins-enriched extracts, ACEs; anthocyanins-free extracts, ACFs) for: (1) total phenolic and anthocyanins contents, (2) free radical (DPPH) scavenging and reactive carbonyl (MGO) species trapping properties, (3) anti-glycation effects (BSA-fructose and BSA-MGO models), (4) anti-Aβ-fibrillation effects (thermal- and MGO-induced), and (5) microglial (BV-2) neuroprotective properties. This is the first study to evaluate the ACFs and ACEs of these six edible berries in this panel of bioassays.

## 2. Results

### 2.1. Total Phenolic and Anthocyanins Contents of Berry Extracts

Each berry crude extract (CE) was fractionated by XAD-16 resin adsorption chromatography as previously reported [25] to yield anthocyanins-free (ACFs) and anthocyanins-enriched (ACEs) extracts after solvent removal in vacuo. The chemical constituents of these berry extracts have been previously characterized by our group using high performance liquid chromatography with ultraviolet (HPLC-UV) and electrospray ionization mass spectrometry (HPLC-ESI-MS) methods and the major phenolic compounds identified were anthocyanins, flavonols, flavanols, ellagitannins, gallotannins, proanthocyanidins, and phenolic acids [26]. In the current study, we confirmed the identities of the individual anthocyanins (as shown in Table 1) in each berry ACEs by conducting HPLC-UV analyses and by comparison of the elution orders and their retention times based on our previous report [26]. The chromatograms for each of the berry ACEs (Supplementary Materials Figures S1A–S6A shown at 520 nm, the characteristic wavelength of anthocyanins), confirmed the presence of anthocyanins (as shown in Table 1) which were in agreement with literature [26]. In addition, the HPLC chromatograms of the berry ACEs were extracted at a wavelength of 280 nm (see Supplementary Materials Figures S1B–S6B) supporting the presence of other phenolic “non-anthocyanin” phytochemicals in the berry ACEs including hydrolyzable and condensed tannins (such as ellagitannins, gallotannins, and proanthocyanidins), flavonols, stilbenes, phenolic acids, etc. Other “non-phenolic” constituents including carbohydrates (oligosaccharides and polysaccharides), vitamins, and minerals have also been reported from several of these berries (see the summary of berry constituents in Supplementary Materials Table S1).

The CEs, ACFs, and ACEs for each berry were evaluated for their total polyphenol contents. As shown in Table 2, the berry ACEs and CEs had polyphenol contents ranging from 5.8–11.3%, and 4.1–9.8%, based on gallic acid equivalents (GAEs), respectively. The berry ACFs had lower polyphenol contents (ranging from 0.3–1.5%). The CEs and ACEs were then further evaluated for their total anthocyanins contents based on cyanidin-3-*O*-glucoside equivalents (Table 2). As expected, the berry ACEs contained higher levels of anthocyanins as compared to their respective CEs with the highest values observed for blackberry (6.3% vs. 2.8%), black raspberry (5.7% vs. 2.8%), and red raspberry (5.6% vs. 4.2%).

### 2.2. Berry Extracts Scavenge Free Radicals in the DPPH Assay

The free radical scavenging capacities of the berry samples were determined using the DPPH assay. Overall, the free radical scavenging capacities of the ACEs and CEs were superior to the ACFs (Table 3). The highest free radical scavenging capacity among the CEs was observed for red raspberry with an IC_50_ value of 268.5 μg/mL while the other CEs had activities with IC_50_ values ranging from 381.1–2865.9 μg/mL. Among the ACEs, blackberry had the highest free radical scavenging activity with an IC_50_ value of 133.8 μg/mL while the other ACEs also showed free radical scavenging activity with IC_50_ values ranging from 337.7–469.8 μg/mL. Among the ACFs, those from blueberry, cranberry, and red raspberry, showed moderate free radical scavenging effects with IC_50_ values ranging from 2010.3–2598.5 μg/mL, which were higher than butylated hydroxytoluene (BHT), and ascorbic acid, used as the positive controls (IC_50_ = 727.8 and 12.3 μg/mL, respectively) [27]. The free radical scavenging activity of these ACFs suggests the presence of “non-anthocyanins phenolic” constituents in these extracts. Indeed, based on previous studies from our laboratory and other research groups, berries are known to contain a wide variety of “non-anthocyanins phenolic” compounds including other flavonoids (flavonols and flavanols), phenolic acids, stilbenoids, hydrolysable tannins (ellagitannins and gallotannins), and condensed tannins (proanthocyanidins) [28,29]. 

### 2.3. Berry Extracts Scavenge Reactive Carbonyl Species (RCS)

The RCS scavenging capacities of the berry extracts (CEs, ACFs, ACEs; all at 100 μg/mL) are presented as the trapping capacity of methylglyoxal (MGO). As shown in Table 3, similar to the DPPH free radical scavenging assay, the ACEs had superior MGO trapping capacity ranging from 16.2–32.8% while the CEs showed RCS trapping capacity ranging from 10.1–21.8%. Moderate MGO-trapping capacity was observed for the ACFs of cranberry, red raspberry, and black raspberry (18.4%, 9.4%, and 8.4%, respectively). The other ACFs showed weak MGO-trapping capacity (up to 3.3%). The positive control, aminoguanidine (AG), a synthetic anti-glycation agent, at equivalent concentration of 100 μg/mL, showed 73.7% MGO-trapping capacity. 

### 2.4. Berry Extracts Inhibit the Formation of AGEs

**Figure 1:**
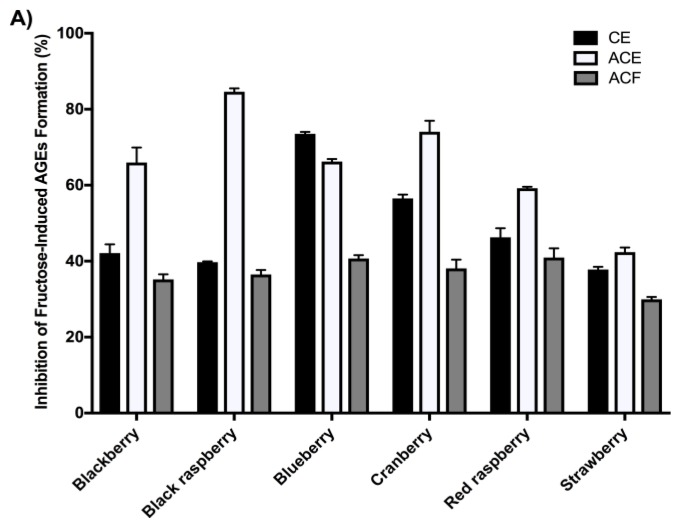

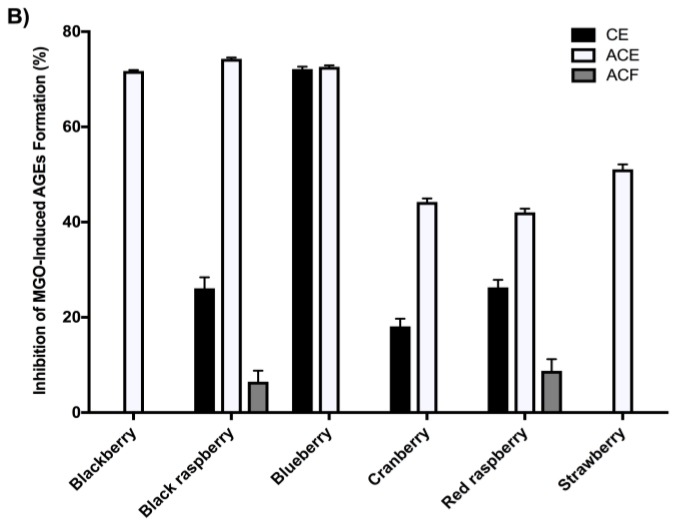
Berry extracts (crude extracts (CEs), anthocyanins-enriched (ACE), and anthocyanins-free (ACF)) were evaluated for their inhibitory effects on the formation of AGEs. The berry extracts were assayed at concentrations of 100 and 500 μg/mL for the BSA-fructose assay (**A**) and BSA-MGO assay (**B**), respectively. Berry extracts were incubated at 37 °C for 21 days for the BSA-fructose assay and 7 days for the BSA-MGO assay. Data are presented as means ± SDs of three independent experiments.

In the fructose-induced AGEs formation assay (Figure 1A), at a concentration of 100 μg/mL, the berry ACEs showed the most potent anti-glycation effects followed by their corresponding CEs and ACFs. Among all of the berry ACEs, black raspberry showed the highest inhibitory effects (85.4%), followed by cranberry (74.5%), blueberry (66.7%), blackberry (66.1%), red raspberry (59.1%), and strawberry (42.8%). In the MGO-induced AGEs formation assay, at the same concentration (100 μg/mL), only the ACEs from black raspberry, blueberry, blackberry, and cranberry showed moderate inhibitory effects of 40.5%, 29.2%, 27.7%, and 25.4%, respectively. Therefore, a higher concentration (500 μg/mL) of these extracts was selected for further evaluation. As shown in Figure 1B, at this higher concentration, the berry ACEs showed the highest inhibitory effects ranging from 42.3–74.6%, followed by their respective CEs and ACFs. Among the ACEs, the most potent anti-glycation effects were from black raspberry (74.8%), blueberry (72.4%) and blackberry (72.2%). The other berry ACEs showed anti-glycation effects ranging from 42.4–51.7% inhibition. 

### 2.5. Berry Anthocyanin-Enriched Extracts (ACEs) Inhibit Aβ Fibrillation

**Figure 2:**
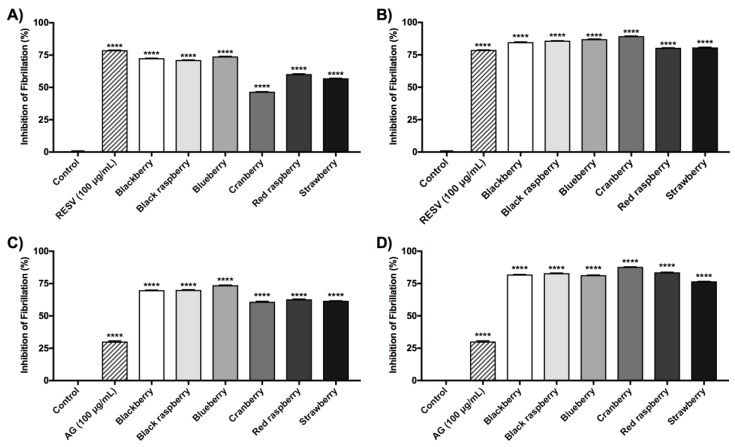
Berry ACEs were evaluated for their inhibitory effects on the fibrillation of Aβ by the ThT assay. The berry ACEs were tested in a thermal-induced Aβ fibrillation model at concentrations of 100 μg/mL (**A**) and 400 μg/mL (**B**) and in a MGO-induced Aβ fibrillation model at concentrations of 100 μg/mL (**C**) and 400 μg/mL (**D**) The Aβ solutions were incubated at 37 °C for 72 h. Resveratrol (RESV; 100 μg/mL) and aminoguanidine (AG; 100 μg/mL) were used as positive controls in the thermal-induced and MGO-induced Aβ fibrillation assays, respectively. Data are presented as means ± SDs of three independent experiments. Statistical significance was reported as the following: *p* ≤ 0.0001 as ****, as compared to the control group.

The berry ACEs had higher phenolic and anthocyanins contents and stronger activities in the free radical scavenging, MGO-trapping, and anti-glycation assays, as compared to their respective CEs and ACFs. Therefore, the berry ACEs were further selected to evaluate their potential neuroprotective effects. The berry ACEs were first evaluated for their anti-Aβ fibrillation effects using two different conditions, thermal-induced and MGO-induced fibrillation of Aβ. In the thermal-induced Aβ fibrillation assay (Figure 2B), all of the berry ACEs (at 400 μg/mL) showed comparable inhibitory effects ranging from 80% to 89%. Cranberry showed the highest activity (89.2%), followed by blackberry (87.8%), black raspberry (86.3%), blueberry (85.1%), strawberry (81.7%), and raspberry (80.4%). Resveratrol (RESV), used as the positive control, showed similar inhibitory effects (79.6%) at a concentration of 100 μg/mL. At the lower test concentration of 100 μg/mL (Figure 2A), the berry ACEs also inhibited Aβ fibrillation ranging from 47–74%. Among all of the berries, blackberry showed the highest inhibitory effect of 74% followed by blueberry (73.6%), and black raspberry (71.5%) which were comparable to RESV (79.7%) at equivalent concentrations of 100 μg/mL. 

A similar trend was observed in the MGO-induced Aβ fibrillation assay as for the thermal-induced assay ( 2C, D). At the higher test concentration of 400 μg/mL, all of the berry ACEs showed similar inhibitory levels ranging from 77–88%. Cranberry, showed the highest inhibitory effect of 88.2%, followed by red raspberry (84.5%), black raspberry (83.1%), blueberry (82.7%), blackberry (82.3%), and strawberry (77.5%). At the lower test concentration of 100 μg/mL, all of the ACEs showed slightly lower inhibitory levels ranging from 61–74%. These inhibitory levels were significantly higher than aminoguanidine (AG; 31.6% inhibition), a synthetic MGO trapping agent, used as a positive control, which was evaluated at an equivalent concentration of 100 μg/mL. 

### 2.6. Berry Anthocyanin-Enriched Extracts (ACEs) Reduce LPS-Induced Inflammation in BV-2 Microglia 

**Figure 3:**
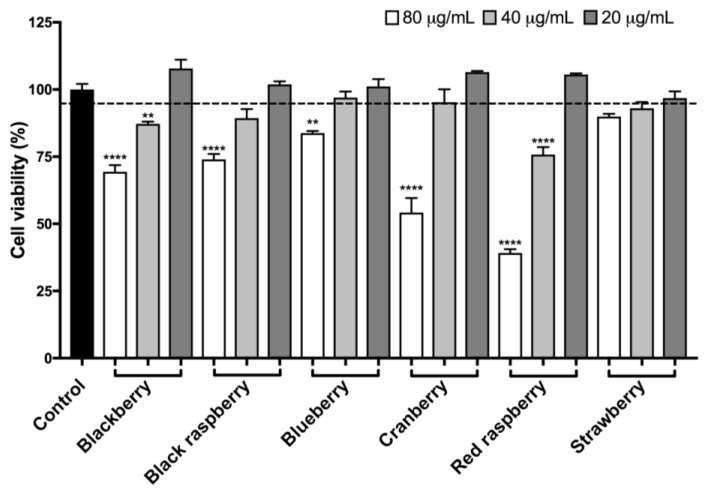
Effects of the berry ACEs (at 20, 40, and 80 μg/mL concentrations) on BV-2 microglia viability using the CTG 2.0 cell viability assay. Statistical significance was reported as *p* ≤ 0.01 as **, and *p* ≤ 0.0001 as ****, as compared to the control group.

While anthocyanins-rich fruits, including açai and blueberry, have been reported to show anti-neuroinflammatory effects in BV-2 microglia [30,31,32], there are limited studies conducted with the other aforementioned edible berries in these cells. Therefore, the berry ACEs were evaluated for potential protective effects against inflammatory stress in BV-2 microglia. The berry ACEs were first evaluated for their effects on BV-2 cell viability at three different concentrations of 20, 40, and 80 μg/mL. As shown in Figure 3, the berry ACEs at the lowest test concentration (20 μg/mL) were non-toxic to the BV-2 cells (cell viability > 95%) and, therefore, this concentration was selected for further biological evaluations.

**Figure 4:**
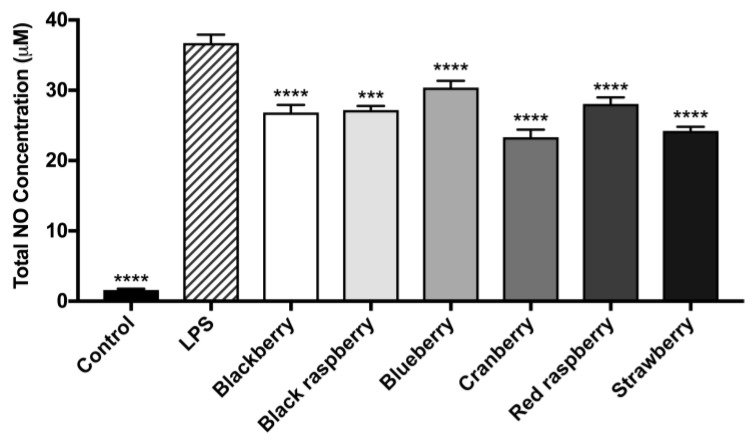
Berry ACEs were evaluated for their inhibitory effects on the production of nitric oxide species (NOS) in murine BV-2 microglia using the Griess reagent. BV-2 microglia were pre-treated with berry ACEs (at 20 μg/mL) for 1 h and inflammatory stress was induced by treatment with lipopolysaccharide (LPS; at 1 μg/mL) for 23 h. Data are presented as means ± SDs with three replicates. Statistical significance was reported as *p* ≤ 0.001 as ***, and *p* ≤ 0.0001 as ****, as compared to the LPS-treated group.

As shown in Figure 4, after induction of inflammation by lipopolysaccharide (LPS), the levels of nitric oxide species (NOS) in LPS treated BV-2 microglia were elevated by 15-fold compared to control cells. All of the berry ACEs (at 20 μg/mL) significantly reduced LPS-induced NOS production in BV-2 microglia. The highest levels of NOS reduction were observed for the ACEs of cranberry (36.5%), strawberry (34.1%), blackberry (27.0%), black raspberry (26.0%), red raspberry (23.6%), and blueberry (17.3%). 

### 2.7. Berry Anthocyanin-Enriched Extracts (ACEs) H_2_O_2_-Induced Oxidative Stress in BV-2 Microglia 

**Figure 5:**
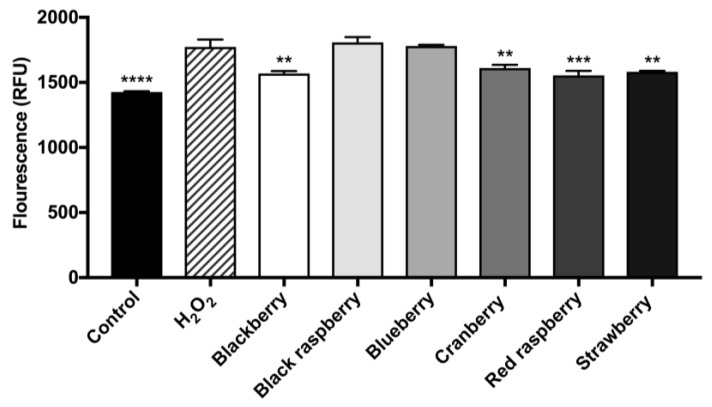
Berry ACEs were evaluated for their inhibitory effects on the production of reactive oxygen species (ROS) in murine BV-2 microglia using the fluorescent probe, DCFDA. BV-2 microglia were incubated with each berry ACEs (at 20 μg/mL) for 24 h and then with media containing the DCFDA reagent (20 μM) for 30 min. Oxidative stress was induced by the treatment with hydrogen peroxide (H_2_O_2_; at 100 μM). Data are presented as means ± SDs with three replicates. Statistical significance was reported as the following: *p* ≤ 0.01 as **, *p* ≤ 0.001 as *** and *p* ≤ 0.0001 as ****, as compared to the H_2_O_2_-treated group.

Next, we evaluated whether the berry ACEs were able to decrease oxidative stress in BV-2 microglia. As shown in Figure 5, when oxidative stress was induced by H_2_O_2_, the levels of reactive oxygen species (ROS) in the H_2_O_2_-treated BV-2 cells increased by 24.2% as compared to control cells. The berry ACEs (at 20 μg/mL) reduced ROS production in the BV-2 microglia up to 12.3% as compared to cells exposed to H_2_O_2_ alone. The ACE of red raspberry showed the highest ROS reduction of 12.3% while the ACEs of blackberry, cranberry, and strawberry showed comparable ROS reduction of 11.5%, 9.2%, and 10.8%, respectively.

### 2.8. Berry Anthocyanin-Enriched Extracts (ACEs) Protect BV-2 Microglia against H_2_O_2_-Induced Cytotoxicity

**Figure 6:**
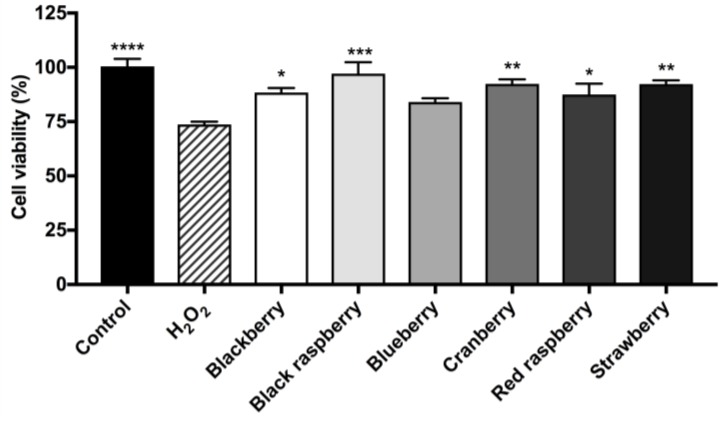
Berry ACEs were evaluated for the protective effects in murine BV-2 microglia. BV-2 microglia were incubated with each berry ACEs (at 20 μg/mL) for 24 h and then cytotoxicity was induced by treatment with hydrogen peroxide (H_2_O_2_; at 100 μM). After 6 h incubation, cellular viability was determined by the CTG 2.0 assay. Data are presented as means ± SDs with three replicates. Statistical significance was reported as the following: *p* ≤ 0.05 as *, *p* ≤ 0.01 as **, *p* ≤ 0.001 as *** and *p* ≤ 0.0001 as ****, as compared to the H_2_O_2_-treated group.

The neuroprotective effects of the berry ACEs were further evaluated in BV-2 microglia against H_2_O_2_-induced cytotoxicity as previously reported [12]. As shown in Figure 6, the cell viability of H_2_O_2_ (100 µM)-treated BV-2 cells was reduced by 26.7% as compared to control cells. After treatment with the ACEs of blackberry, black raspberry, blueberry, cranberry, red raspberry, and strawberry (at 20 µg/mL), the viabilities of H_2_O_2_ challenged BV-2 microglia were increased by 14.7%, 23.4%, 10.4%, 18.7%, 13.8%, and 18.6%, respectively, as compared to the H_2_O_2_ treatment group. 

### 2.9. Berry Anthocyanin-Enriched Extracts (ACEs) Decrease H_2_O_2_-Induced Activity of Caspase-3/7 in BV-2 Microglia

**Figure 7:**
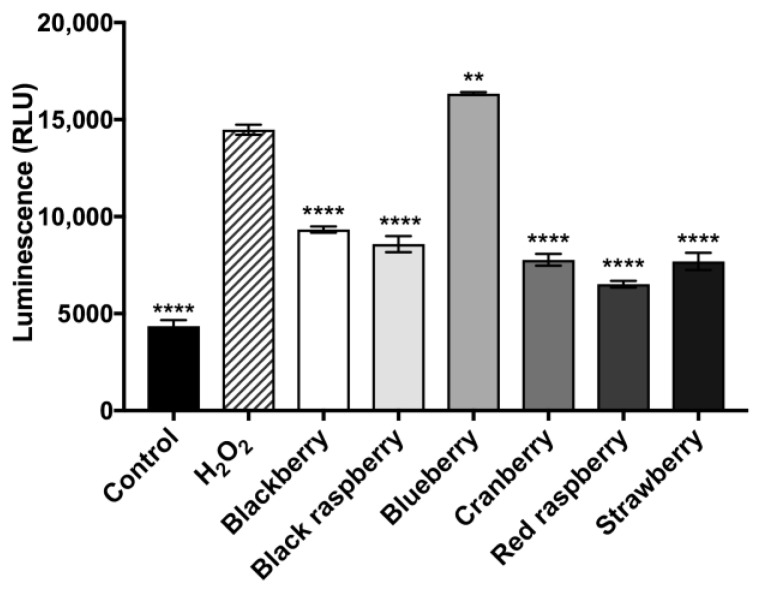
Berry ACEs were evaluated for their inhibitory effects on caspase-3/7 activity in BV-2 microglia treated with hydrogen peroxide (H_2_O_2_; at 100 μM). Cells were pre-treated with 20 μg/mL of berry ACEs for 24 h, then treated with H_2_O_2_ for 6 h. Data are presented as means ± SDs with three replicates. Statistical significance was reported as *p* ≤ 0.01 as **, *p* ≤ 0.0001 as ****, as compared to the H_2_O_2_-treated group.

Microglia death induced by oxidative stress is highly associated with a group of cysteinyl-aspartate-specific proteases including caspase-3/7 [33]. To further evaluate whether the microglia neuroprotective effects of the berry ACEs are mediated by regulation of caspase-3/7, we measured caspase-3/7 activity in BV-2 cells treated with the berry ACEs. As shown in Figure 7, the activity of caspase-3/7 in BV-2 cells exposed to oxidative stress (by treatment of H_2_O_2_ at 100 μM) increased by 69.9% as compared to the control group. The ACEs for blackberry, black raspberry, cranberry, red raspberry, and strawberry (at concentrations of 20 μg/mL) significantly decreased caspase-3/7 activities by 35.5%, 40.7%, 46.3%, 54.9%, and 46.9%, respectively, as compared to the H_2_O_2_ treatment group.

## 3. Discussion 

Alzheimer’s disease (AD) is a neurodegenerative disorder linked with oxidative damage and inflammation and despite considerable research into understanding the pathology of this disease, there are still no drugs which can reverse its progression. Natural products, including polyphenolic-enriched extracts from functional foods, show promise as dietary agents for the prevention and potential management of AD [34,35,36]. Published data suggest that the neuroprotective effects of berry fruits are associated with their polyphenolic compounds, particularly, anthocyanins [37,38]. For instance, anthocyanins have been reported to protect against inflammatory and oxidative stress mediated neuroinflammation and neurodegeneration in adult mouse cortex [39], as well as in brains of postnatal rats [40], and rats fed a high-fat diet [41]. In light of these data, we designed the current study to evaluate the potential neuroprotective effects of polyphenolic-enriched extracts of six common edible berries, namely, blackberry, black raspberry, blueberry, cranberry, red raspberry, and strawberry. To investigate whether anthocyanins were indeed the major contributors to the neuroprotective effects of these berries, we prepared anthocyanins-free (ACFs) and anthocyanins-enriched (ACEs) extracts from each crude berry extract for biological evaluation. 

Overall, the berry ACEs had higher total polyphenol and anthocyanins contents and showed superior antioxidant, MGO trapping, and anti-glycation activities compared to their respective CEs and ACFs. Notably, when protein glycation was induced by MGO, a reactive AGEs precursor, the berry ACEs showed superior inhibitory effects against the formation of AGEs (Figure 1B) compared to their respective CEs and ACFs, which was in agreement with their superior MGO trapping capacity (Table 3). Anthocyanins, including delphinidin-3-rutinoside and cyanidin-3-*O*-rutinoside from blackcurrant berry extract have been previously reported to trap MGO by forming anthocyanins-mono-MGO adducts [42]. Similarly, the anthocyanin, cyanidin-3-*O*-rutinoside, has also been reported to inhibit MGO-induced glycation via its carbonyl trapping ability [43]. Therefore, the superior activities of the ACEs compared to their respective CEs and ACFs suggested that anthocyanins were indeed major contributors to the overall biological activities of these berries. 

While the overall trend for most of the berries showed superior anti-AGE effects of their ACEs compared to their respective CEs and ACFs, blueberry CE was slightly more active than its ACE in the fructose-BSA assay. This suggests that other “non-anthocyanin phenolic” constituents in the blueberry CE, such as catechins and proanthocyanidins, could also possibly contribute to its overall anti-glycation activity as has been previously reported [44]. Also, in general, the berry ACFs were less active in the MGO-trapping and anti-glycation assays as compared to their respective ACEs. However, it is interesting to note that the ACF of cranberry also showed inhibition of fructose-induced AGEs formation (39.0%) and MGO-trapping capacity (18.4%) suggesting that there are ‘non-anthocyanins’ constituents in this berry which may also play a role in its biological effects. Indeed, it has previously been reported that procyanidin oligomers present in water fractions of cranberry show anti-glycation and MGO trapping activities [45]. In addition, our group has also reported that oligosaccharides isolated from an aqueous extract of cranberry showed inhibitory effects on the formation of AGEs by scavenging free radicals and not through an MGO-trapping mechanism [10]. Therefore, although anthocyanins may play an important role in the observed biological activities of the berry extracts in this selected panel of bioassays, other non-anthocyanin constituents, such as found in blueberry and cranberry, may also contribute to the overall activities of these fruits through additive, complementary, and/or synergistic effects. 

Given that the berry ACEs were superior in the above assays as compared to their respective ACFs and CEs, these samples were selected for further biological evaluation in AD-based assays. The berry ACEs were first assessed for their inhibitory effects on the fibrillation of Aβ peptides, the abnormal accumulation and deposition of which is regarded as a diagnostic hallmarker of AD. We used the ThT binding assay to assess both thermal-induced (using resveratrol as positive control) and MGO-induced (using aminoguanidine as a positive control) fibrillation of Aβ. As shown in Figure 2, all of the berry ACEs showed anti-Aβ fibrillation activities in a concentration dependent manner at 100 and 400 µg/mL. In the thermal-induced Aβ fibrillation assay, the berry ACEs showed comparable activity as resveratrol at an equivalent concentration of 100 µg/mL (Figure 2A). However, in the MGO-induced Aβ fibrillation assay, the berry ACEs showed superior activities to aminoguanidine at an equivalent concentration of 100 µg/mL (Figure 2C Although our observations with the berry ACEs are in agreement with published reports on the known Aβ fibrillation inhibitory effects of polyphenols [46,47], a major limitation of the current study lies in the test concentrations used in these in vitro experiments which are not translatable to the in vivo situation. The bioavailability of anthocyanins is generally low [48,49] although their levels in berry fruits can range from 10 s to 100 s of milligrams per serving [48].

Next, we evaluated the potential neuroprotective effects of the berry ACEs in cellular based assays. The effects of the berry ACEs on murine BV-2 microglia was assessed and a non-cytotoxic concentration (20 µg/mL; Figure 3) was selected for further evaluation. At this concentration, the berry ACEs decreased LPS-induced production of NOS and H_2_O_2_-induced production of ROS in BV-2 microglia (Figure 4 and Figure 5, respectively). This suggested that the berry ACEs reduced inflammatory and oxidative stresses in BV-2 microglia which is in agreement with similar effects reported for anthocyanins from other berries such as açai and blueberry [30,31,32]. Apart from their anti-inflammatory effects, their antioxidant effects may also be another plausible mechanism that contributes to the overall microglial neuroprotection of the berry ACEs in BV-2 cells. Indeed, the berry ACEs reduced the production of cellular ROS and increased the viability of BV-2 cells (Figure 5 and Figure 6, respectively), suggesting that the berry ACEs were able to ameliorate oxidative stress induced damage in BV-2 cells. This effect was further supported by examination of a cysteinyl-aspartate protease, namely, caspase-3/7, in BV-2 cells (Figure 7). Caspase-3/7 serves as an executioner protease when cells are undergoing apoptosis via the intrinsic (oxidative stress) or extrinsic (ligand–death receptor) pathways. During this process, caspase-3/7 is able to cleave a variety of proteins associated with cell DNA repairing and proliferation. Therefore, our findings showed that the berry ACEs were able to maintain BV-2 cell viability against oxidative stress by downregulation of caspase-3/7 activity. This is in agreement with previous studies where other phenolics have also been reported to show neuroprotective effects including reducing the oxidative stress induced cell death of neurons via modulation of caspase-3 activity [50]. These data suggest that these common edible berries may have potential neuroprotective effects and that their anthocyanins contribute, in part, towards these biological effects. However, the structural diversity of the various anthocyanins found in these berries and the possible synergistic effects among themselves, as well as with other “non-anthocyanin” phytochemicals may lead to differences in their biological effects [51]. For instance, anthocyanin metabolism and bioavailability have been reported to be influenced by differences in their chemical structure including the type of aglycone and their attached moieties [51]. Therefore, the diverse structural varieties of the different anthocyanins present in these six edible berries (see Figures S1A–S6A in the Supplementary Materials) could indeed influence their potential neuroprotective effects. Also, anthocyanins have been reported to have synergistic effects with other ‘non-anthocyanin’ phenolic compounds in their biological properties and the possibility of other substances such as vitamins, minerals, and carbohydrates (oligosaccharides and polysaccharides) in these berry extracts (see the summary of berry constituents in Supplementary Materials Table S1) contributing toward their potential neuroprotective effects cannot be excluded. Lastly, given that our evaluations were conducted using biophysical and in vitro assays, which are not translatable to the in vivo situation, further studies using animal models are warranted to evaluate the potential neuroprotective effects of these berries against AD [52,53]. 

## 4. Materials and Methods 

### 4.1. Chemicals 

Aminoguanidine hydrochloride (AG), bovine serum albumin (BSA), d-fructose, d-ribose, 2,3-dimethylquinoxaline (DQ), gallic acid (GA), resveratrol (RESV), butylated hydroxytoluene (BHT), 2,2-diphenyl-1-picrylhydrazyl (DPPH), methylglyoxal (MGO), 1,2-phenylenediamine (PD), hydrogen peroxide (H_2_O_2_), trifluoroacetic acid (TFA), lipopolysaccharide (LPS), 2’,7’-dichlorofluorescin diacetate reagent (DCFDA), XAD-16 Amberlite resin, and HPLC-grade methanol were purchased from Sigma-Aldrich Chemical Co. (St. Louis, MO, USA). Beta amyloid 1-42 (Aβ) was purchased from AnaSpec Inc. (Fremont, CA, USA). Griess reagent was purchased from Promega Corp. (Madison, MI, USA). 

### 4.2. Berry Materials 

Blackberry and black raspberry freeze dried powders were provided by Scenic Fruit Company (Gresham, OR, USA) and Berri Products LLC (Corvallis, OR, USA), respectively. Strawberry freeze dried powder was provided by the California Strawberry Commission (Watsonville, CA, USA). Freeze dried red raspberry fruit was provided by the Washington Red Raspberry Commission from HoneyVille Farms (Brigham City, UT, USA). Cranberry fruit powder was provided by Ocean Spray Cranberries Inc. (Middleborough, MA, USA). Blueberry fresh fruit was purchased from a local Stop & Shop grocery store (Narragansett, RI, USA). 

### 4.3. Preparation and Fractionation of Berry Extracts 

Our laboratory has previously reported on the preparation and chemical characterization of phenolic-enriched extracts from berries. [25,26,54,55,56] Therefore following these previously published protocols, each berry sample (100 g for freeze dried berry powders; 250 g for blueberry fresh fruit) was extracted with methanol (500 mL) to yield crude extracts (CEs) after solvent removal in vacuo. The dried methanolic berry CEs were fractionated to yield anthocyanins-free (ACFs) and anthocyanin-enriched (ACEs) extracts using XAD-16 Amberlite resin adsorption chromatography. Briefly, each berry CE (10 g) was reconstituted in distilled water (200 mL) and adsorbed on to an XAD-16 Amberlite resin column (300 mm × 3.5 mm, i.d.). After 4 h of adsorption time, the column was eluted with distilled water (1 L) followed by methanol (1 L) to yield ACFs and ACEs, respectively, for each berry, after solvent removal in vacuo. Based on our previously published study [12], and data from our preliminary experiments, the concentrations of berry extracts used for the biophysical assays were as follows: 100 µg/mL for the MGO trapping assay; 100 and 500 μg/mL for the BSA-fructose assay and BSA-MGO assay, respectively; 100 and 400 μg/mL for the ThT Aβ fibrillation assay.

### 4.4. Total Phenolic and Anthocyanins Content 

The total phenolic content for each berry sample (CEs, ACFs, ACEs) was determined using the Folin–Ciocalteau method and expressed as gallic acid equivalents (GAEs) as previously reported [12]. Briefly, the berry samples (10–20 mg/mL) were diluted 1:100 with methanol/H_2_O (1:1, *v*/*v*). 100 μL of each sample was incubated with 50 μL of Folin–Ciocalteau reagent for 5 min at room temperature (25 °C). 150 μL Na_2_CO_3_ and 250 μL H_2_O were added to each sample and incubated at 40 °C in the dark for 30 min. Samples were then cooled on ice to room temperature and centrifuged. The absorbance was determined at 756 nm on a Spectramax M2 plate reader operated by SoftmaxPro v.4.6 software (Molecular Devices, Sunnyvale, CA, USA). The total anthocyanins content was performed using the pH differential method (expressed as cyanidin-3-*O*-glucoside equivalents) as previously reported [57]. Briefly, each berry sample was made to a stock solution (20 mg/mL). Two aliquots (1.0 mL) of the stock solution were placed into 25 mL volumetric flasks which were then filled up to mark using pre-prepared buffers of pH 1.0 or pH 4.5, respectively. The absorbance of these buffer solutions were recorded at 510 and 700 nm, respectively, using a SpectraMax M2 plate reader (Molecular Devices, Sunnyvale, CA, USA). 

### 4.5. 2,2-Diphenyl-1-Picrylhydrazyl (DPPH) Free Radical Scavenging Assay 

The free radical scavenging capacities of the berry samples (CEs, ACFs, ACEs) were determined by the 2,2-diphenyl-1-picrylhydrazyl (DPPH) free radical scavenging assay as previously reported [9]. The assay was performed in a 96-well plate using serial dilutions of 100 μL aliquots of each of the berry samples (ranging from 0.2–5 mg/mL). DPPH solution (100 μL) was added to each well, and the plate was incubated at room temperature in the dark for 30 min. The absorbance was determined at 517 nm using a SpectraMax M2 plate reader. 

### 4.6. Reactive Carbonyl Species (Methylglyoxal; MGO) Trapping Assay 

The trapping capacity of reactive carbonyl species (RCS) by the berry samples (CEs, ACFs, ACEs) was evaluated using the previously reported method [6]. Briefly, a reaction solution consisting of MGO (5 mM), PD (derivatization reagent, 20 mM), and DQ (internal standard, 5 mM) were freshly prepared in 0.1 M phosphate buffer, pH 7.4. The MGO solution (0.25 mL) was mixed with phosphate buffer (0.25 mL) serving as a blank solution. Each berry sample (0.25 mL of 100 µg/mL) was added to MGO (0.25 mL of 5 mM). After incubation at 37 °C for 1 h, PD and DQ (0.125 mL, each), were added to each mixture. The solutions were then left at room temperature for 30 min. The amount of 2-methylquinoxaline (2-MQ), the derivative of residual MGO, was quantified by HPLC-DAD as previously reported [6]. 

### 4.7. Inhibition of the Formation of Advanced Glycation Endproducts (AGEs) 

The inhibition of formation of AGEs was determined using the BSA-fructose and BSA-MGO intrinsic fluorescence methods as previously reported [7]. All solutions were prepared in 0.1 M phosphate buffer of pH 7.4. Reaction mixtures consisted of 10 mg/mL of BSA and 100 mM of d-fructose or 5 mM of MGO. Each berry samples (CEs, ACFs, and ACEs) were evaluated at low and high concentrations of 100 and 500 μg/mL, respectively. For the BSA-fructose assay, the samples were incubated at 37 °C for 21 days while for the BSA-MGO assay, the samples were incubated for 7 days. After their respective incubation periods, all samples were transferred to a 96-well fluorescence reading plate and the intrinsic fluorescence levels were measured at an excitation wavelength of 360 nm and an emission wavelength of 430 nm using a Spectramax M2 plate reader. 

### 4.8. Anti-Aβ Fibrillation Assay 

The inhibitory effects on Aβ fibrillation by the berry ACEs were determined by the thioflavin T (ThT) assay following previously reported methods with minor modifications [12]. Two protocols, thermal-induced Aβ fibrillation and MGO-induced Aβ fibrillation, were followed. The final concentrations of Aβ were adjusted to 50 µM. The berry ACEs were evaluated at low and high concentrations of 100 and 400 µg/mL, respectively. For the thermal-induced assay, Aβ solutions were incubated at 37 °C for 72 h while for the MGO-induced assay, 1 mM MGO was added to the reaction mixture prior to incubation. Resveratrol (RESV; at 100 µg/mL) and aminoguanidine (AG; at 100 µg/mL) served as the positive controls for the thermal-induced and MGO-induced assays, respectively. After 72 h, 20 µL of each Aβ solution was mixed with 100 µL of 10 µM ThT solution and the intrinsic fluorescence was determined on a SpectraMax M2 plate reader at excitation and emission wavelengths of 450 and 490 nm, respectively.

### 4.9. Cell Culture 

Murine BV-2 microglia were a kind gift from Grace Y. Sun (University of Missouri at Columbia, MO, USA). Cells were maintained in 10% heat inactivated FBS, with 1% P/S (100 U/mL penicillin, 100 mg/mL streptomycin) in high glucose (4.5 g/L) DMEM/F-12 media at 37 °C in 5% CO_2_. The berry ACEs were dissolved in distilled water to obtain stock solutions (10 mg/mL) and then diluted with serum free media to yield final concentrations of 20 μg/mL. Control groups were treated with DMSO (≤0.1%) in serum free media. 

### 4.10. Effects of Berry ACEs on BV-2 Microglia Viability

The effects of each berry ACE on BV-2 microglia viability were determined by the Cell Titer Glo (CTG 2.0) assay (Promega; Fitchburg, WI, USA) as previously reported by our laboratory [12]. In brief, BV-2 cells were plated in 96-welled white wall, clear bottom plates at a density of 100,000 cells/mL in DMEM/F-12 media. After a period of 24 h, medium was removed and replaced with each berry ACE treatment (at 20, 40 and 80 μg/mL) for 24 h. Then cellular viability of BV-2 cells was assessed with CTG 2.0 method by measuring luminescence with a SpectraMax M2 Plate Reader. 

### 4.11. Measurement of Total Nitric Oxide Species (NOS) in BV-2 Microglia by Griess Assay 

The production of total nitric oxide species (NOS) in BV-2 microglia was evaluated by the Greiss reagent assay kit (Promega, Fitchburg, WI, USA) as previously reported by our laboratory [58]. Briefly, BV-2 microglia were seeded in clear 24-well plates at 100,000 cells/mL in DMEM/F-12 media. Cells were pre-treated with berry ACEs (at 20 μg/mL) for 1 h. Next, BV-2 microglia were exposed to lipopolysaccharide (LPS; at 1 μg/mL) for 23 h. Culture media was then transferred to a 96-well plate and measured for total NOS.

### 4.12. Measurement of Reactive Oxygen Species (ROS) in BV-2 Microglia 

Reactive oxygen species (ROS) in BV-2 microglia was determined using a fluorescent probe, dichlorofluorescin diacetate (DCFDA), as previously reported by our laboratory [59]. BV-2 microglia were seeded in black walled clear bottom 96-well plates at 100,000 cells/mL in DMEM/F-12 media. The BV-2 microglia were incubated with each berry ACEs (at 20 μg/mL) for 24 h and then media from the treatment groups was replaced with media containing DCFDA (20 μM). After incubation for 30 min, the DCFDA media was removed and cells were washed. Next, the BV-2 microglia were treated with hydrogen peroxide (H_2_O_2_; at 100 μM) and then incubated for 6 h. The amounts of ROS in the BV-2 cells were measured using a SpectraMax M2 Plate Reader at excitation and emission wavelengths of 495 and 529 nm, respectively.

### 4.13. Measurement of Caspase-3/7 Activity in BV-2 Microglia after H_2_O_2_ Exposure

Caspase-3/7 activity was determined using a Caspase-Glo 3/7 assay kit (Promega, Fitchburg, WI, USA) as previously described by our laboratory [60]. BV-2 microglia were pre-treated for 24 h with each berry ACEs (at 20 μg/mL). Cells were then treated with hydrogen peroxide (H_2_O_2_; at 100 μM) for 6 h. After incubation, Caspase-Glo 3/7 assay was performed according to the manufacturer’s protocol.

### 4.14. Measurement of BV-2 Microglia Viability after Exposure to Hydrogen Peroxide (H_2_O_2_) 

Cell viability was determined using the Cell Titer Glo (CTG 2.0) assay (Promega; Fitchburg, WI, USA) as previously reported by our laboratory [12]. Briefly, BV-2 microglia were seeded in white walled clear bottom 96-well plates at a density of approximately 100,000 cells/mL in DMEM/F-12 media. After incubation of 24 h for adhesion, the BV-2 microglia were treated with the berry ACEs (at 20 μg/mL) for 24 h. Cell toxicity was then induced by exposure of the cells to hydrogen peroxide (H_2_O_2_; at 100 μM). After 6 h incubation, cellular viability was determined by the CTG 2.0 assay on a SpectraMax M2 Plate Reader. Cell viability was reported as a percent of vehicle control (DMSO).

### 4.15. Statistical Analysis 

The DPPH assay, MGO trapping assay, anti-glycation, and ThT assays were performed in at least three replicates for each sample. In the fluorescence reading assays (anti-glycation and ThT assays), the inhibition rate (% inhibition) was defined using the following equation: % inhibition = [1 − (fluorescence intensity of solution with treatment/fluorescence intensity of control solution)] × 100%. The cellular assays were conducted as three independent experiments each with three replicates per sample and the values presented herein were chosen from one of these experiments with three replicates for each sample. Data were reported as mean ± standard deviation (SD) with an *n* = 3. Statistical significance was determined using One-Way Analysis of Variance with corrections for multiple comparisons using Dunnett’s Test using Graphpad Prism Software (La Jolla, CA, USA). 

## 5. Conclusions

In summary, six common edible berries including blackberry, black raspberry, blueberry, cranberry, red raspberry, and strawberry were evaluated in a panel of bioassays for their potential anti-glycation and neuroprotective effects. The berry CEs, ACFs, and ACEs showed free radical scavenging, MGO-trapping, and anti-glycation effects. Given the higher phenolic contents and biological effects of the berry ACEs in these assays, they were selected for further evaluation for their potential neuroprotective effects in the anti-Aβ fibrillation and BV-2 microglia bioassays. The berry ACEs inhibited the fibrillation of Aβ in both thermal- and MGO-induced models, reduced LPS- and H_2_O_2_-induced inflammatory and oxidative stresses in BV-2 microglia, increased BV-2 cell viability against H_2_O_2_–induced oxidative stress induced cytotoxicity, and decreased the activity of an apoptotic protease (capase-3/7) in BV-2 cells. These data suggest that these common edible berries may have potential microglial neuroprotective effects and that their anthocyanins contribute, in part, towards these biological effects. However, given that these studies were conducted using biophysical and in vitro assays, which are not translatable to the in vivo situation, further studies using in vivo models are warranted to evaluate the potential effects of these berries against AD. 

## Figures and Tables

**Table 1:** Anthocyanins reported in the six edible berry extracts.

Berry Sample	Anthocyanins
Blackberry	cyanidin-3-*O*-glucoside, cyanidin-3-*O*-arabinoside, cyanidin-3-*O*-xyloside, cyanidin-3-*O*-malonylglucoside, and cyanidin-3-*O*-dioxalylglucoside
Black raspberry	cyanidin-3-*O*-sambuoside, cyanidin-3-*O*-glucoside, cyanidin-3-*O*-xylosylrutinoside, and cyanidin-3-*O*-rutinoside
Blueberry	cyanidin-3-*O*-galactoside, petunidin-3-*O*-galactoside, petunidin-3-*O*-glucoside, peonidin-3-*O*-galactoside, and malvidin-3-*O*-glucoside
Cranberry	cyanidin-3-*O*-galactoside, cyanidin-3-*O*-arabinoside, peonidin-3-*O*-galactoside, and peonidin-3-*O*-arabinoside
Red raspberry	cyanidin-3-*O*-glucoside, cyanidin-3-*O*-arabinoside, and delphinidin-3-*O*-arabinoside
Strawberry	cyanidin-3-*O*-glucoside, pelargonidin-3-*O*-glucoside, and pelargonidin-3-*O*-rutinoside

**Table 2:** Berry extracts and their total phenolic and total anthocyanins contents.

Common Name	Species	Family	Extract ^a^	Phenolic Content ^b^	Anthocyanins Content ^c^
Blackberry	*Rubus* sp.	Rosaceae	CE	6.8%	2.8%
			ACE	11.3%	6.3%
			ACF	0.3%	n.d.^d^
Black raspberry	*Rubus occidentalis*	Rosaceae	CE	4.1%	2.8%
			ACE	7.5%	5.7%
			ACF	0.3%	n.d.
Blueberry	*Vaccinium angustifolium*	Ericaceae	CE	9.8%	2.5%
			ACE	8.5%	4.4%
			ACF	1.5%	n.d.
Cranberry	*Vaccinium macrocarpon*	Ericaceae	CE	7.7%	1.2%
			ACE	6.4%	3.8%
			ACF	1.3%	n.d.
Red raspberry	*Rubus idaeus*	Rosaceae	CE	6.4%	4.2%
			ACE	5.8%	5.6%
			ACF	0.9%	n.d.
Strawberry	*Fragaria ananassa*	Rosaceae	CE	3.8%	2.2%
			ACE	7.3%	3.7%
			ACF	0.8%	n.d.

^a^ CE: crude extract; ACE: anthocyanins-enriched extract; ACF: anthocyanins-free extract; ^b^ value expressed as in *w*/*w*% of gallic acid equivalents; ^c^ value expressed as in *w*/*w*% of cyanidin-3-*O*-glucoside equivalents; ^d^ n.d. = not detected.

**Table 3:** Free radical scavenging capacity (DPPH assay) and reactive carbonyl species (MGO) scavenging capacity of berry extracts.

Berry	Extracts	Free Radical Scavenging Capacity (IC_50_; µg/mL)	MGO Trapping Capacity (%)
Blackberry	CE	1968.6 ± 22.3	13.7
	ACE	133.8 ± 11.1	30.3
	ACF	n.d. ^a^	3.3
Black raspberry	CE	2865.9 ± 62.8	21.8
	ACE	409.6 ± 23.7	31.1
	ACF	n.d.	8.4
Blueberry	CE	381.1 ± 3.1	15.6
	ACE	454.3 ± 4.6	29.2
	ACF	2598.5 ± 34.7	n.d.
Cranberry	CE	392.6 ± 2.9	10.1
	ACE	434.5 ± 7.1	32.8
	ACF	2217.3 ± 11.1	18.4
Red raspberry	CE	268.5 ± 8.6	13.7
	ACE	337.7 ± 1.6	18.2
	ACF	2010.3 ± 60.2	9.4
Strawberry	CE	n.d.	14.5
	ACE	469.8 ± 2.5	16.2
	ACF	n.d.	n.d.
BHT ^b^		727.8 ± 11.6	n.t.^d^
Ascorbic acid ^b^		12.3 ± 1.7	n.t.
AG ^c^		n.t.	73.7

^a^ n.d. = not detected; ^b^ Butylated hydroxytoluene (BHT) and ascorbic acid, positive controls for DPPH assay; ^c^ Aminoguanidine (AG), positive control for MGO trapping assay; ^d^ n.t. = not tested.

## Supplementary Materials

Supplementary materials can be found at http://www.mdpi.com/1422-0067/19/2/461/s1.

## Abbreviations

AGEs	advanced glycation endproducts
Aβ	beta amyloid
AD	Alzheimer’s disease
ACE	anthocyanins-enriched extract
ACF	anthocyanins-free extract
AG	aminoguanidine
BHT	butylated hydroxytoluene
BSA	bovine serum albumin
CE	crude extract
GAE	gallic acid equivalent
LPS	lipopolysaccharide
MGO	methylglyoxal
NOS	nitric oxide species
RCS	reactive carbonyl species
ROS	reactive oxygen species
