# Supplementary material for: Evaluation of Polyphenol Anthocyanin-Enriched Extracts of Blackberry, Black Raspberry, Blueberry, Cranberry, Red Raspberry, and Strawberry for Free Radical Scavenging, Reactive Carbonyl Species Trapping, Anti-Glycation, Anti-β-Amyloid Aggregation, and Microglial Neuroprotective Effects
Source: Int J Mol Sci. 2018 Feb 3;19(2):461. doi: 10.3390/ijms19020461 (PMC5855683; doi:10.3390/ijms19020461)
Supplement: Supplementary file 1 [file ijms-19-00461-s001.pdf]

# Supplementary Materials

**Evaluation of Polyphenol Anthocyanin-Enriched Extracts of Blackberry, Black Raspberry, Blueberry, Cranberry, Red Raspberry, and Strawberry for Free Radical Scavenging, Reactive Carbonyl Species Trapping, Anti-Glycation, Anti-β-Amyloid Aggregation, and Microglial Neuroprotective Effects**

Hang Ma [1,2,3†], Shelby L. Johnson [2,3†], Weixi Liu [4], Nicholas A. DaSilva [2,3], Susan Meschwitz [5], Joel A. Dain [4], and Navindra P. Seeram [2,3*]

[1]School of Chemical and Environment Engineering, Wuyi University; International Healthcare Innovation Institute (Jiangmen), Jiangmen, Guangdong 529020, China; hang_ma@uri.edu (H.M.)

[2]Bioactive Botanical Research Laboratory, Department of Biomedical and Pharmaceutical Sciences, College of Pharmacy, University of Rhode Island, Kingston, RI 02881, USA; shelby_johnson@uri.edu (S.L.J.); ndasilva@my.uri.edu (N.A.D.)

[3]George and Anne Ryan Institute for Neuroscience, University of Rhode Island, Kingston, RI 02881, USA

[4]Department of Chemistry, University of Rhode Island, Kingston, RI 02881, USA; weixi_liu@my.uri.edu (W.L.); jdain@chm.uri.edu (J.A.D.)

[5]Department of Chemistry, Salve Regina University, Newport, RI 02840, USA; Susan.Meschwitz@salve.edu (S.M.)

*Correspondence: nseeram@uri.edu (N.P.S); Tel.: +1-401-874-9367

[†]These authors contributed equally to this work.

## List of contents

**HPLC-DAD analyses of phenolics in the berry ACEs** …….…….………...…….……….….…...………3

**Figure S1.** HPLC-DAD chromatogram of blackberry ACE ……………..…………………….….…….…..4

**Figure S2.** HPLC-DAD chromatogram of black raspberry ACE ………..……………………....……...5

**Figure S3.** HPLC-DAD chromatogram of blueberry ACE ………..…………...………………..…..…...6

**Figure S4.** HPLC-DAD chromatogram of cranberry ACE ………………..……………………...……...7

**Figure S5.** HPLC-DAD chromatogram of red raspberry ACE ………………………………..…..……...8

**Figure S6.** HPLC-DAD chromatogram of red strawberry ACE …………..……………………..….…...9

**Table S1.** Chemical constituents of six berries……………………………………………………..……..10

**HPLC-DAD analyses of phenolics in the berry ACEs**

Each berry ACE was analyzed by HPLC-DAD as previously reported by our laboratory for the presence of phenolics (at 280 nm) and anthocyanins (at 520 nm) (Figures S1-S6). Briefly, each berry ACEs (dissolved in DMSO; all at equivalent concentrations of 10 mg/mL) were analyzed on a Luna C18 column (250 × 4.6 mm i.d., 5 μM; Phenomenex) with a flow rate of 0.75 mL/min and injection volume of 20 μL for each sample. A linear gradient solvent system consisting of solvent A (0.1% aqueous trifluoroacetic acid) and solvent B (methanol) at room temperature was used as follows: 0–30 min, 10% to 60% B; 30–35 min, 60% to 100% B; 35–40 min, 100% B; 40–41 min, 100% to 10% B; 41–51 min, 10% B.

**Figure S1**

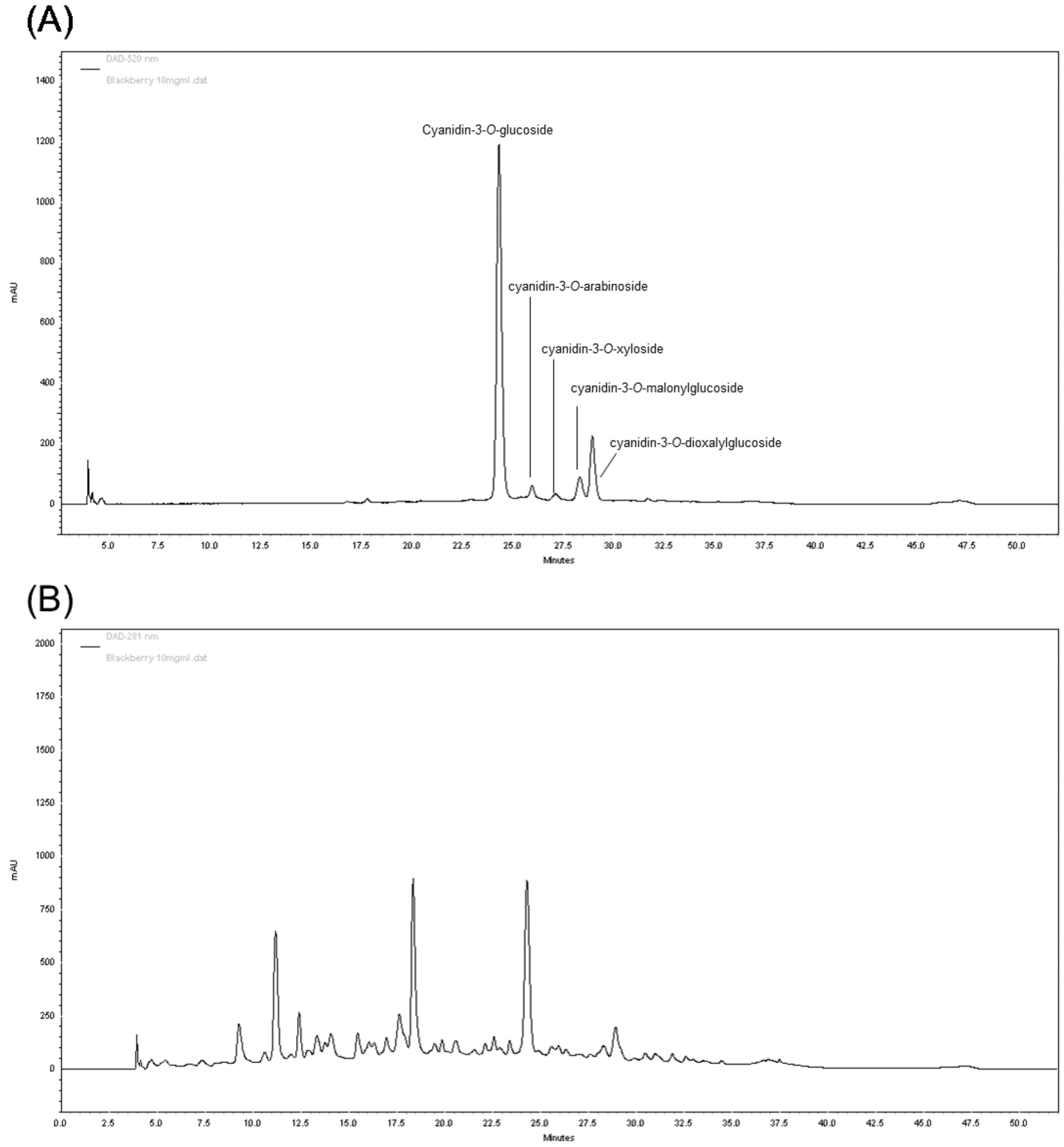


**Figure S1.** HPLC-DAD chromatogram of blackberry ACE monitored at (A) wavelength of 520 nm showing its major anthocyanins as cyanidin-3-*O*-glucoside, cyanidin-3-*O*-arabinoside, cyanidin-3-*O*-xyloside, cyanidin-3-*O*-malonylglucoside, and cyanidin-3-*O*-dioxalylglucoside; and (B) wavelength of 280 nm showing the presence of phenolics.

**Figure S2**

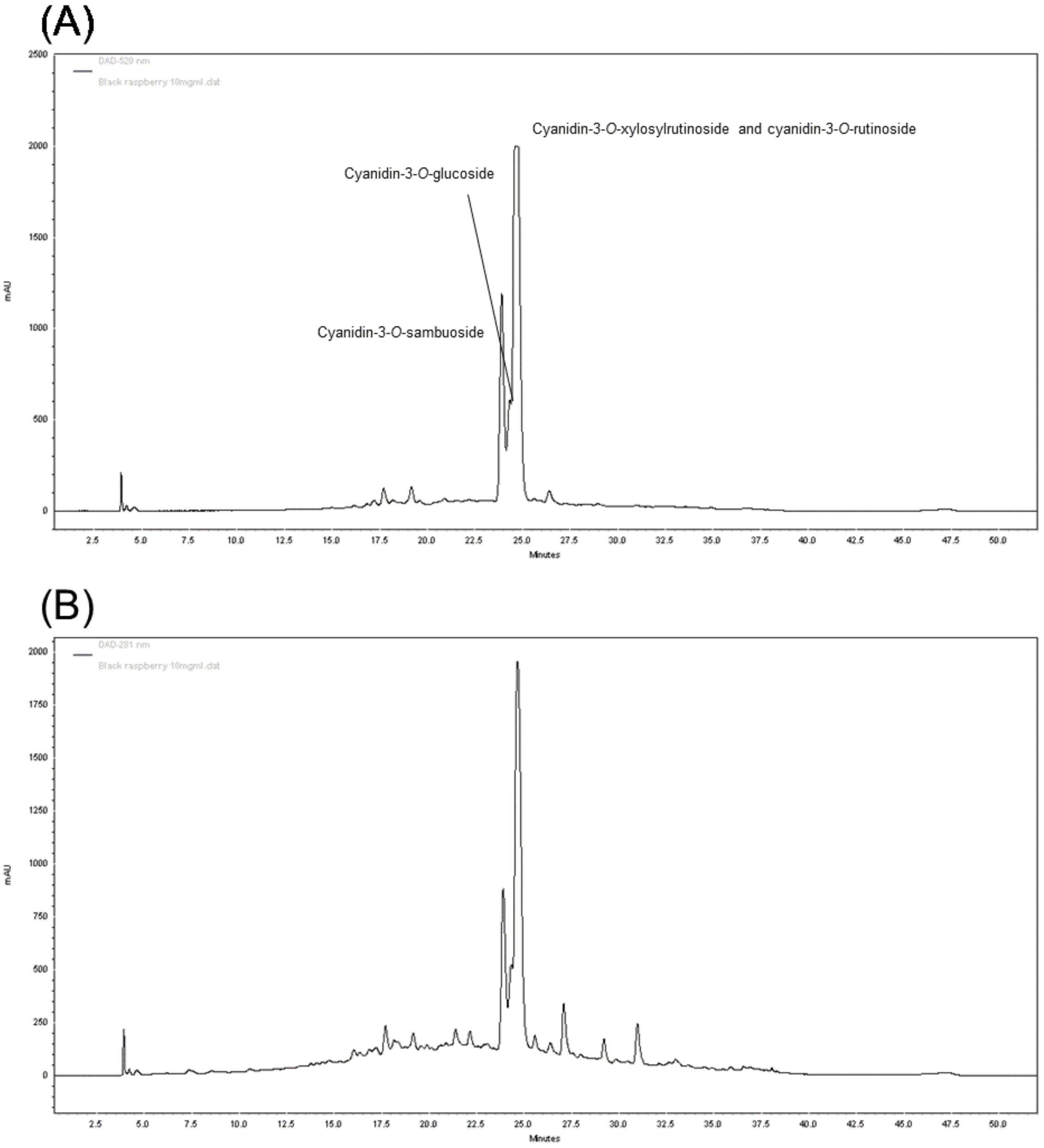


**Figure S2.** HPLC-DAD chromatogram of black raspberry ACE monitored at (A) wavelength of 520 nm showing its major anthocyanins as cyanidin-3-*O*-sambuoside, cyanidin-3-*O*-glucoside, cyanidin-3-*O*-xylosylrutinoside, and cyanidin-3-*O*-rutinoside; and (B) wavelength of 280 nm showing the presence of phenolics.

**Figure S3**

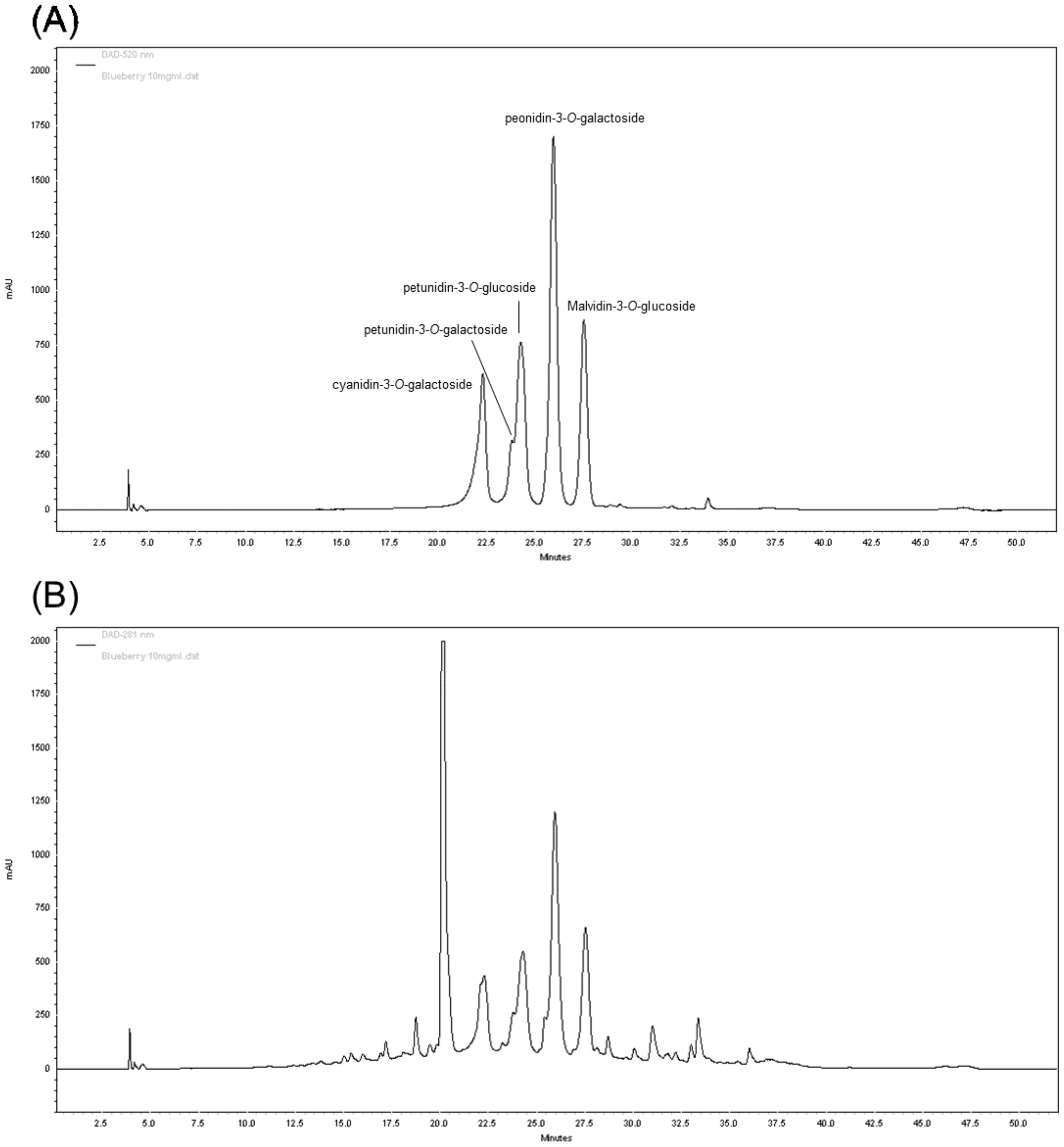


**Figure S3.** HPLC-DAD chromatogram of blueberry ACE monitored at (A) wavelength of 520 nm showing its major anthocyanins as cyanidin-3-*O*-galactoside, petunidin-3-*O*-galactoside, petunidin-3-*O*-glucoside, peonidin-3-*O*-galactoside, and malvidin-3-*O*-glucoside; and (B) wavelength of 280 nm showing the presence of phenolics.

**Figure S4**

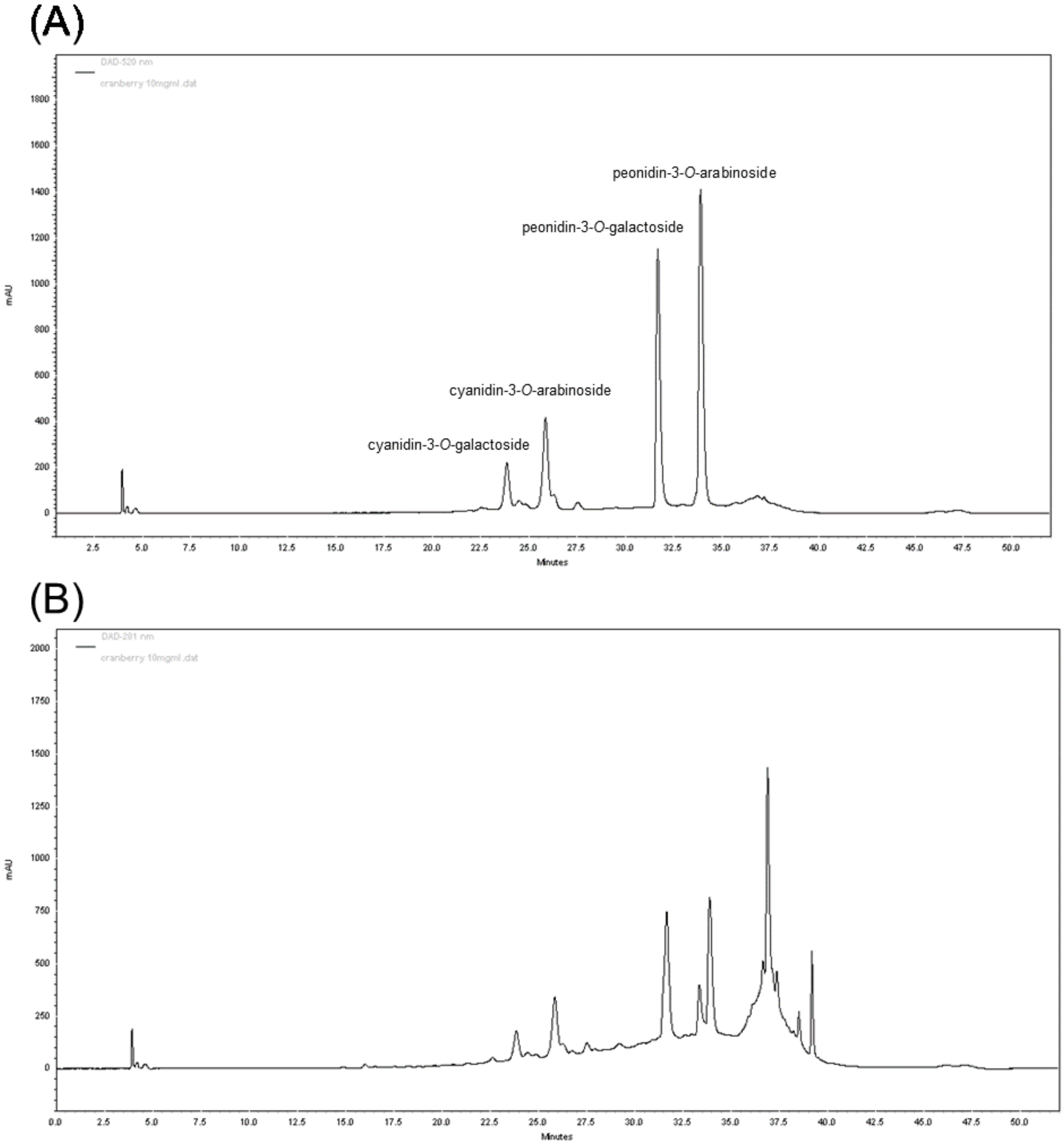


**Figure S4.** HPLC-DAD chromatogram of cranberry ACE monitored at (A) wavelength of 520 nm showing the identity of its major anthocyanins as cyanidin-3-*O*-galactoside, cyanidin-3-*O*-arabinoside, peonidin-3-*O*-galactoside, and peonidin-3-*O*-arabinoside; and (B) wavelength of 280 nm showing the presence of phenolics.

**Figure S5**

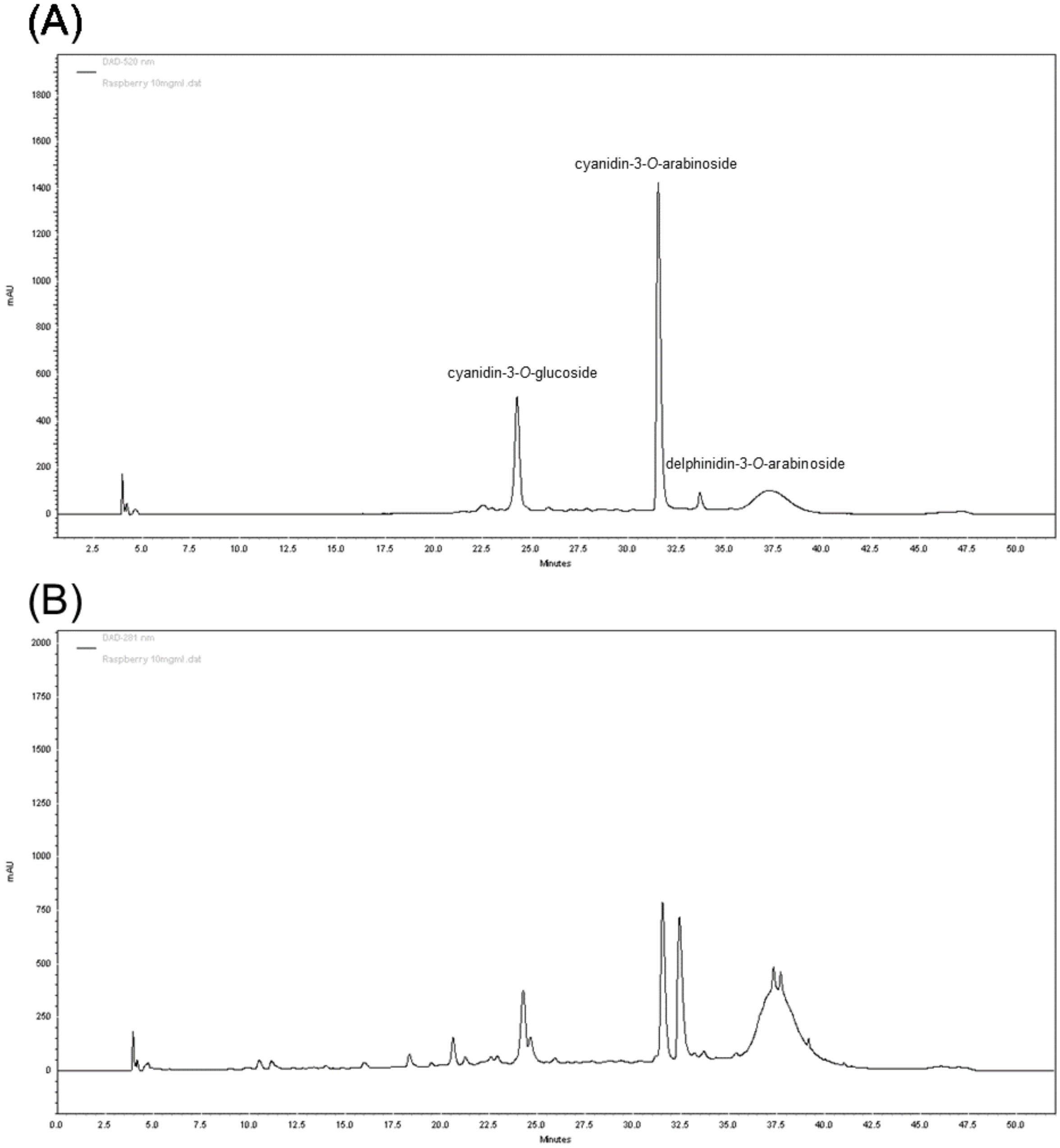


**Figure S5.** HPLC-DAD chromatogram of red raspberry ACE monitored at (A) wavelength of 520 nm showing its major anthocyanins as cyanidin-3-*O*-glucoside, cyanidin-3-*O*-arabinoside, delphinidin-3-*O*-arabinoside; and (B) wavelength of 280 nm showing the presence of phenolics.

**Figure S6**

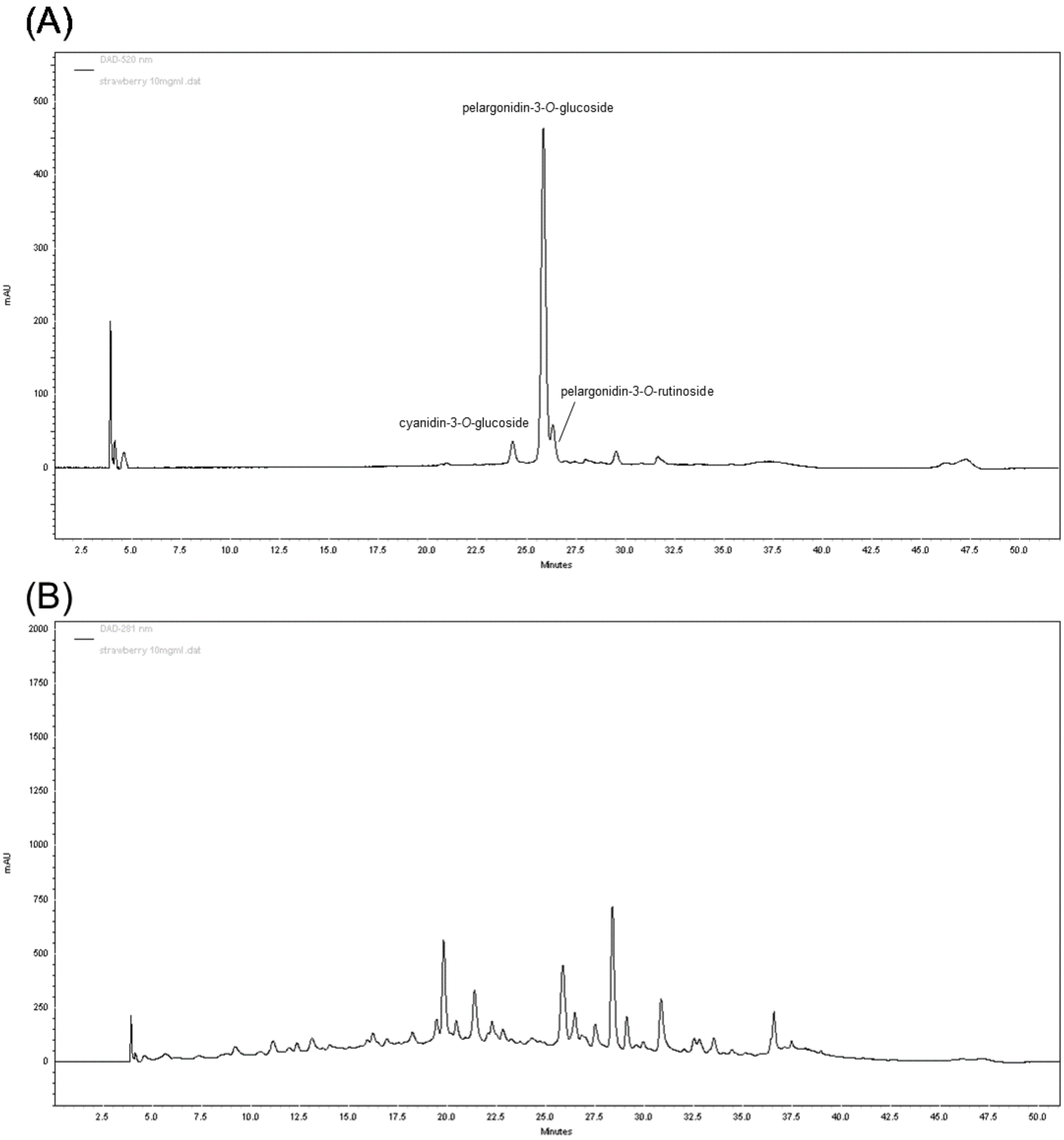


**Figure S6.** HPLC-DAD chromatogram of red raspberry ACE monitored at (A) wavelength of 520 nm showing its major anthocyanins as cyanidin-3-*O*-glucoside, pelargonidin-3-*O*-glucoside, and pelargonidin-3-*O*-rutinoside; and (B) wavelength of 280 nm showing the presence of phenolics.

**Table S1**. Chemical constituents including non-phenolic and phenolic 'non-anthocyanin' compounds of blackberry, black raspberry, blueberry, cranberry, red raspberry, and strawberry.

| Berry | Non-phenolic compounds | Phenolic compounds (non-anthocyanins) | Reference |
|---|---|---|---|
| Blackberry | Carbohydrates, organic acids, vitamins, minerals, etc. | Ellagitannins, flavonols, phenolic acids and procyanidins, lignans etc. | [1-4] |
| Black raspberry | Carbohydrates, organic acids, minerals, folic acid, and β-sitosterol, etc. | Ellagitannins, ellagic acid, flavonols, ferulic acid, etc. | [1, 3-5] |
| Blueberry | Carbohydrates, organic acids, minerals, vitamins, etc. | Proanthocyanidins, flavonols, catechins, stilbenes, etc. | [1, 3, 4, 6-8] |
| Cranberry | Carbohydrates, organic acids, vitamins, minerals, etc. | Proanthocyanidins, flavonols, stilbenes, etc. | [1, 3, 4, 9-11] |
| Red raspberry | Carbohydrates, organic acids, vitamins, minerals, etc. | Ellagitannins, gallotannins, ellagic acid, flavonols, catechins, etc. | [1, 3, 4, 12] |
| Strawberry | Carbohydrates, organic acids, vitamins, minerals, etc. | Ellagitannins, ellagic acid, flavonols, phenolic acids, etc. | [1, 3, 4] |

**References:**


1. Seeram, N. P.; Adams, L. S.; Zhang, Y.; Lee, R.; Sand, D.; Scheuller, H. S.; Heber, D., Blackberry, black raspberry, blueberry, cranberry, red raspberry, and strawberry extracts inhibit growth and stimulate apoptosis of human cancer cells in vitro. *J. Agric. Food Chem.* **2006,** *54*, 9329-9339.
2. Kaume, L.; Howard, L. R.; Devareddy, L., The blackberry fruit: a review on its composition and chemistry, metabolism and bioavailability, and health benefits. *J. Agric. Food Chem.* **2012,** *60*, 5716-5727.

3. Mikulic-Petkovsek, M.; Schmitzer, V.; Slatnar, A.; Stampar, F.; Veberic, R., Composition of sugars, organic acids, and total phenolics in 25 wild or cultivated berry species. *J. Food Sci.* **2012,** *77*, C1064-1070.
4. Esteban, M. A.; Villanueva, M. J.; Lissarrague, J., Effect of irrigation on changes in berry composition of Tempranillo during maturation. Sugars, organic acids, and mineral elements. *Am. J. Enol. Vitic* **1999,** *50*, 418-434.
5. Tulio, A. Z., Jr.; Reese, R. N.; Wyzgoski, F. J.; Rinaldi, P. L.; Fu, R.; Scheerens, J. C.; Miller, A. R., Cyanidin 3-rutinoside and cyanidin 3-xylosylrutinoside as primary phenolic antioxidants in black raspberry. *J. Agric. Food Chem.* **2008,** *56*, 1880-1888.
6. Sun, X.; Liu, N.; Wu, Z.; Feng, Y.; Meng, X., Anti-tumor activity of a polysaccharide from blueberry. *Molecules* **2015,** *20*, 3841-3853.
7. Morikawa, C.; Saigusa, M., Mineral composition and accumulation of silicon in tissues of blueberry (*Vaccinum corymbosus cv*. Bluecrop) cuttings. *Plant Soil* **2004,** *258*, 1-8.
8. Chen, C.; Li, Y.; Xu, Z., Chemical principles and bioactivities of blueberry. *Acta Pharm. Sinica* **2010,** *45*, 422-429.
9. Sun, J.; Liu, W.; Ma, H.; Marais, J. P.; Khoo, C.; Dain, J. A.; Rowley, D. C.; Seeram, N. P., Effect of cranberry (*Vaccinium macrocarpon*) oligosaccharides on the formation of advanced glycation end-products. *J. Berry Res.* **2016,** *6*, 149-158.
10. Seeram, N. P.; Adams, L. S.; Hardy, M. L.; Heber, D., Total cranberry extract versus its phytochemical constituents: antiproliferative and synergistic effects against human tumor cell lines. *J. Agric. Food Chem.* **2004,** *52*, 2512-2517.
11. Lacombe, A.; Wu, V. C.; Tyler, S.; Edwards, K., Antimicrobial action of the American cranberry constituents; phenolics, anthocyanins, and organic acids, against Escherichia coli O157: H7. *Int. J. Food Microbiol.* **2010,** *139*, 102-107.
12. Mullen, W.; McGinn, J.; Lean, M. E.; MacLean, M. R.; Gardner, P.; Duthie, G. G.; Yokota, T.; Crozier, A., Ellagitannins, flavonoids, and other phenolics in red raspberries and their contribution to antioxidant capacity and vasorelaxation properties. *J. Agric. Food Chem.* **2002,** *50*, 5191-5196.
